# Management of plexiform neurofibromas in neurofibromatosis type 1: An Italian Delphi consensus

**DOI:** 10.1007/s10072-026-09290-z

**Published:** 2026-09-23

**Authors:** Claudia Santoro, Elena Arkhangelskaya, Ambra Bonvicino, Irene Bruno, Mario Cirillo, Maria Cristina Diana, Marica Eoli, Flavio Facchini, Franco Ionna, Chiara Leoni, Marina Macchiaiolo, Giuseppe Marulli, Vittoria Nazzi, Sergio Paolini, Eva Trevisson, Martino Ruggieri, Veronica Saletti

**Affiliations:** 1 Department of Woman, Child, and General and Specialized Surgery, University of Campania “Luigi Vanvitelli”, Napoli, Italy; 2 Radiology Unit, IRCCS Istituto Giannina Gaslini, Genova, Italy; 3 Dermatology Unit, Città della Salute e della Scienza, Torino, Italy; 4 Department of Pediatrics, Institute for Maternal and Child Health Burlo Garofolo, Trieste, Italy; 5 Department of Advanced Medical and Surgical Sciences, University of Campania “Luigi Vanvitelli”, Naples, Italy; 6 Pediatric Neurology and Neuromuscular Disorders Unit, IRCCS Giannina Gaslini Institute, Genoa, Italy; 7 Unit of Molecular Neuro-Oncology, Member of the European Reference Network Genturis, Fondazione IRCCS Istituto Neurologico Carlo Besta, Milano, Italy; 8 Plastic Surgery, Meyer Children’s Hospital, Firenze, Italy; 9 Dipartimento di Medicina e Scienze della Salute “V. Tiberio” della Università degli studi del Molise, Oncologia Chirurgia Cervico-Facciale, Pineta Grande Hospital Castel Volturno CE, Castel Volturno, Italy; 10 Department of Woman and Child Health and Public Health, Center for Rare Diseases and Birth Defects, Fondazione Policlinico Universitario A. Gemelli IRCCS, Roma, Italy; 11 Rare Diseases and Medical Genetic Unit, Bambino Gesù Children’s Hospital, Roma, Italy; 12 Department of Biomedical Sciences, Humanitas University, Milan, Italy; 13 Thoracic Surgery, Unit - IRCCS Humanitas Research Hospital, Milan, Italy; 14 Department of Neurosurgery, Fondazione IRCCS Istituto Neurologico Carlo Besta, Milano, Italy; 15 Department of Neurosurgery, IRCCS Neuromed, Pozzilli, Isernia, Italy; 16 Department of Women’s and Children’s Health, Clinical Genetics Unit, University of Padova, Padova, Italy; 17 Unit Pediatric Clinic, Department of Clinical and Experimental Medicine, AOU “Policlinico”, PO “G. Rodolico”, University of Catania, Catania, Italy; 18 Department of Pediatric Neuroscience, Member of the European Reference Network Genturis, Fondazione IRCCS Istituto Neurologico Carlo Besta, Celoria 11 street, Milano, 20121 Italy

**Keywords:** Neurofibromatosis type 1, Plexiform neurofibroma, Delphi method, Consensus

## Abstract

**Background:**

Plexiform neurofibromas (PNs) are among the most complex and challenging manifestations of neurofibromatosis type 1 (NF1). Recent advances in understanding their epidemiology, natural history, biology, and imaging features, together with the introduction of MEK inhibitors, have significantly reshaped clinical management. Despite availability of international recommendations for tumor surveillance in NF1, several aspects of PN management remain incompletely defined in clinical practice.

**Methods:**

A multidisciplinary panel of 17 Italian NF1 experts conducted a Real-Time Delphi consensus process including a literature review, thematic analysis, and development of consensus statements. Key domains encompassed diagnosis, imaging assessment, surveillance, surgical evaluation, multidisciplinary management, and treatment of PNs. Two rounds of voting were conducted through a dedicated online platform, ensuring anonymity, real-time feedback, and iterative refinement of the statements.

**Results:**

Consensus was achieved on 49 final statements. Key recommendations emphasized the importance of early identification of PNs, assessment of internal tumor burden through whole-body MRI, standardized imaging acquisition and reporting, timely recognition and characterization of distinct nodular lesions, and implementation of risk-adapted surveillance strategies. The panel also underscored the importance of patient education regarding signs suggestive of malignant transformation, and recommended a structured multidisciplinary approach to clinical decision-making. Agreement was additionally reached on practical aspects including radiological follow-up, criteria for surgical referral, definitions of PN-related symptoms, inoperability, and integration of surgical and targeted treatment strategies.

**Conclusions:**

This national consensus provides an evidence-informed and clinically oriented framework for management of NF1-associated PNs. Complementing existing international recommendations, it offers practical guidance in areas where evidence remains limited.

## Key points

- Early diagnosis of Plexiform Neurofibromas (PNs) in patients with Neurofibromatosis type 1 (NF1) is essential to optimize surveillance and therapeutic decision-making.
- Whole-body MRI at transition to adulthood is useful for assessing internal tumor burden and guiding risk-adapted management strategies.
- Distinct nodular lesions should be accurately reported in MRI reports and require dedicated multidisciplinary assessment and follow-up.
- Patient education is a fundamental component of PN management and supports early recognition of disease progression.
- Multidisciplinary team evaluation is essential for the management of complex, symptomatic or potentially symptomatic PNs.
- Surgery and MEK inhibitors should be considered complementary therapeutic strategies within a multidisciplinary framework.
- This national multidisciplinary consensus provides practical recommendations to support clinical decision-making in NF1-associated PNs.

## Introduction

Neurofibromatosis type 1 (NF1) is one of the most common autosomal dominant disorders, affecting approximately 1 in 3,000 live births worldwide.

It is a complex multisystem disorder with a wide range of age-dependent clinical manifestations and a significant predisposition to neurological and non-neurological tumors [1].

NF1 is caused by heterozygous pathogenic variants in the *NF1* gene leading to loss of neurofibromin function and subsequent increased RAS activity and activation of the RAF/MEK/ERK pathway, which plays a central role in tumor pathogenesis [1].

Among the NF1 diagnostic criteria revised in 2021, plexiform neurofibromas (PNs) represent one of the most challenging. PNs are benign peripheral nerve sheath tumors that can arise from any cranial nerve (except the optic nerve), as well as from peripheral nerves, and they occur in up to 50% of patients with NF1 [2].

Cranial and peripheral nerves are composed of axons ensheathed by myelin produced by Schwann cells and embedded within perineurial and mesenchymal cells. PNs primarily originate from Schwann cells and are highly vascular, multicellular tumors that grow along a nerve and its branches, often forming large masses, particularly within the brachial or lumbosacral plexuses [3]. The term *plexiform* derives from the Latin word plexus, meaning “network,” reflecting the characteristic complex and highly interconnected growth pattern of these tumors along multiple nerves, branches, or fascicles.

According to the 2021 World Health Organization (WHO) classification of Central Nervous System tumors and the 2020 WHO classification of Soft Tissue and Bone tumors, PNs belong to a heterogeneous group of neuroectodermal origin soft tissue tumors associated with nerve structures [4, 5] including other benign tumors such as neurofibroma, schwannoma, perineurioma, and hybrid peripheral nerve sheath tumor, as well as malignant tumors such as malignant peripheral nerve sheath tumor (MPNST), epithelioid MPNST, malignant melanotic nerve sheath tumor, and neuroendocrine tumor of the cauda equina [4–6].

Over the past two decades, substantial progress has been made in understanding the epidemiology, natural history, biology, and imaging characteristics of PNs, which, although benign, can significantly impact the quality of life (QoL) through pain, neurological deficits, functional impairment secondary to compression of adjacent structures, and physical disfigurement [7]. Furthermore, PNs have the potential to develop into MPNSTs, which are linked to a poor prognosis [8, 9].

The recent introduction of MEK inhibitors for symptomatic and inoperable PNs has profoundly reshaped the therapeutic landscape, previously limited to symptom management and surgical resection [10–12]. The opportunity to initiate treatment during childhood, together with recent advances in the understanding of PNs, have prompted a comprehensive reassessment of clinical practice. This reassessment encompasses the definition of optimal timing and diagnostic strategies, as well as the refinement of criteria used to classify PNs as symptomatic and inoperable.

In addition, recent insights into the molecular mechanisms driving progression toward malignant transformation, together with the radiologic identification of a subset of PNs termed distinct nodular lesions (DNLs), characterized by accelerated growth and potentially more aggressive behavior, have led to a re-evaluation of surveillance strategies and the role of prophylactic surgery [9, 13, 14].

In this evolving and complex scenario, clinical decision-making for NF1-associated PNs remains heterogeneous across medical specialties, centers, and countries [15]. Given this variability and the persistence of areas of uncertainty in clinical practice, the need for standardized clinical guidance has become increasingly evident [16].

To address this gap, a Delphi consensus process was conducted with a multidisciplinary panel of Italian experts in NF1 and PN management. This initiative aimed to identify areas of agreement and divergence in current clinical practice and formulate consensus-based recommendations to support multidisciplinary clinical decision-making in managing PNs across different age groups and clinical scenarios.

## Methods

### Study design and development

#### Delphi methodology

The Delphi method is a reliable measurement instrument commonly used across settings, including health-related areas, to reach consensus on a topic through a structured group of experts [17]. The conventional Delphi method is based on multiple rounds of questionnaires aimed at collecting and synthesizing opinions and has four main characteristics: anonymity, iteration, controlled feedback, and statistical aggregation of group responses [18]. However, the multi-round Delphi (MRD) method is characterized by some shortfalls, including the length of the process. Recently, the Real Time Delphi (RTD) method has emerged as an efficient-centered approach. It allows an enhancement of the overall process, as it is characterized by greater effectiveness, does not require iterative rounds of an online survey, and the responses are recorded and updated in “real time”. Recent studies revealed comparable outcomes for the MRD and the RTD surveys and highlighted the advantages of RTD in terms of time saving and achievement of better convergence on final scoring [19].

#### Study coordination

This RTD survey was led and administered by Helaglobe (Florence, Italy), a team of experts in consensus methodologies that was responsible, in collaboration with two of the panelists (CS and VS) (identified as project coordinators) for literature review, organization and coordination of the expert group, development and analysis of questionnaires, and drafting of the final report of the survey.

#### Expert panel selection

Panelists were selected among clinicians working in high-volume referral centers for NF1, each managing an annual caseload of at least 100 patients. To ensure a comprehensive perspective across the disease spectrum, clinicians were chosen based on their expertise in the management of both pediatric and adult patients with NF1 and PN. Attention was paid to achieving broad geographical representation, including centers from Northern, Central, and Southern Italy, in order to reflect regional variability and differences in healthcare organization and clinical practice. Participating clinicians also identified surgeons and radiologists with whom they routinely collaborate in the multidisciplinary management of patients with PN, thereby ensuring the inclusion of specialists with substantial disease-specific expertise and established clinical experience.

#### Consensus statement development

The recommendations included in this consensus are based on a combination of published evidence and expert opinion. Statements addressing well-established aspects of PN diagnosis and surveillance are supported by a relatively robust body of literature and international recommendations, whereas other areas, including surveillance intervals, criteria for inoperability, and surgical decision-making, rely predominantly on expert consensus due to the limited availability of high-quality prospective studies.

*Step 1*,* Desk analysis*: A working group composed of the coordinators (CS and VS) and Helaglobe drafted a preliminary list of principles and research questions following a literature review on PN management in NF1. Predefined keywords were used for literature search in the main biomedical PubMed/Medline database, and the search string was as follows: “((“"neurofibromatosis 1"“[MeSH Terms] OR “"neurofibromatosis 1"“[All Fields] OR “"neurofibromatosis type 1"“[All Fields]) AND (“"neurofibroma, plexiform"“[MeSH Terms] OR (“"neurofibroma"“[All Fields] AND “"plexiform"“[All Fields]) OR “"plexiform neurofibroma"“[All Fields] OR “"neurofibroma plexiform"“[All Fields])) AND ((y_10[Filter]) AND (ffrft[Filter]) AND (humans[Filter]) AND (english[Filter]))”.

The following selection/inclusion criteria were used during the literature review:

- Studies in English;
- Published in the last 10 years;
- Limited to humans;
- Major publications with greater scientific evidence (meta-analysis, systematic reviews, clinical trials, clinical studies, guidelines, reviews, real-world data) relevant to the topic of the RTD study.

The literature search was complemented by the inclusion of landmark publications and international recommendations considered relevant to the topic, irrespective of publication date.

*Step 2*,* Statements definition and meeting of the expert board*: Following the literature review process, the coordinators (CS and VS) developed 57 statements related to 9 principal domains: selection of patients to be included in the diagnostic and monitoring process, NF1-associated PNs diagnosis, MRI-based techniques for the assessment and measures of PNs, MRI-based classification of PNs, MRI classification of DNLs, natural history of PNs, morbidity and mortality of PNs, time of surveillance of PNs, surgical assessment of PNs.

The 57 statements were subsequently reviewed and discussed during an online meeting involving Helaglobe and the coordinators, together with 15 additional experts selected according to the predefined expert panel selection criteria. The Italian NF1 Expert Panel (I-NF1-EP) consisted of adult and pediatric neurologists (ME, VS), pediatricians (CS, IB, MCD, MM, CL, MR), geneticists (ET), dermatologists (AB), radiologists (EA), and surgeons (GM, VN, FI, MC, FF, SP). Patient advocacy groups (PAGs), including ANF (Associazione Nazionale Neurofibromatosi), LINFA ODV (Associazione Lottiamo Insieme contro la Neurofibromatosi), and ANANAS APS (Associazione Nazionale Aiuto per la Neurofibromatosi Amicizia e Solidarietà), were consulted during this phase and provided feedback on the proposed statements from the patient and caregiver perspective. Their contribution informed the discussion process; however, they were not involved in the Delphi voting procedure.

**Fig. 1:**
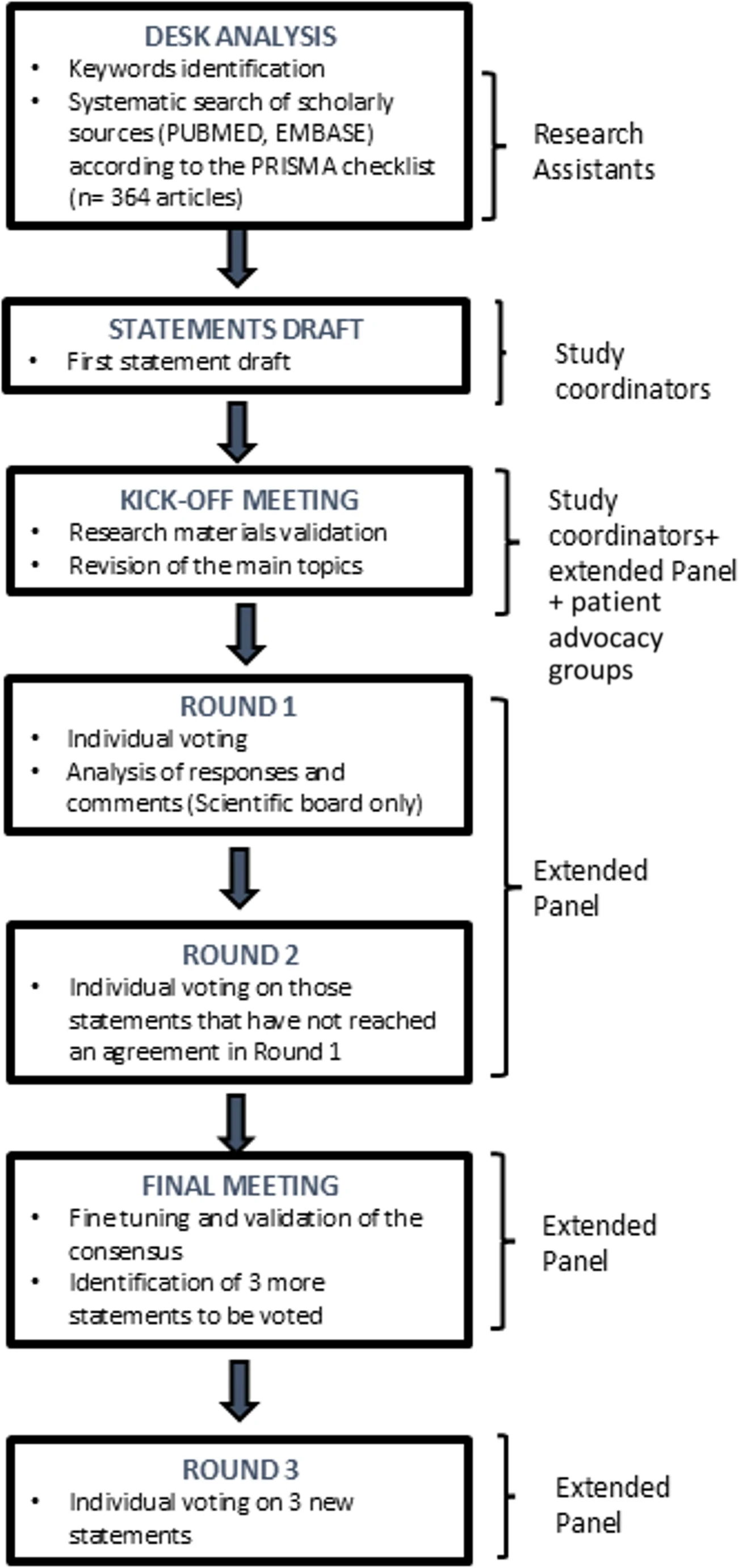
Flow chart of the study

*Step 3*,* Online voting phases and meetings of the Delphi panel*: The 17-member I-NF1-EP, (comprising the two project coordinators and 15 additional experts), was invited by email to participate in the RTD. All materials, including the survey, were in English language. The voting panel expressed a judgment on 57 statements defined during Step 2, described above. The RTD survey was conducted on a proprietary platform developed by Helaglobe. Participants were provided with personal access to the online portal for three weeks, between June and July 2025. By accessing the portal during the process, the panelists were able to vote and comment on each statement. They could also revise their responses multiple times within the allocated time, with real-time calculation of agreement and feedback. Individual answers and comments remained anonymous and were visible only to the moderators. After the initial voting stage, based on the panelists’ comments, the working group decided to merge some statements, resulting in a total of 49 statements to be voted on in the next phase. After the second meeting and discussion, a third phase of voting was necessary for the surgery items: three new statements were uploaded on the online platform, and voting was allowed between the end of July and the first week of August 2025 (Fig. 1).

*Step 4*,* Data analysis*: Voting was undertaken by the Delphi panelists using a 5-point Likert scale to indicate the level of agreement on each statement: 1 = absolutely disagree, 2 = disagree, 3 = neither agree nor disagree, 4 = agree, 5 = strongly agree. The answers were expressed as percentage responses for each statement. A total cumulative agreement was defined as the sum of the response percentages for items 4 (‘agree’) and 5 (‘strongly agree’).

Consensus for each statement was reached if at least 75% of the Delphi panelists ranked the statement ≥ 4 on the 5-point Likert scale described above. This level of total cumulative agreement was set as the median threshold to define the consensus, consistent with several other studies in the healthcare setting [20].

#### Ethical considerations

This study did not involve patients, patient data, biological materials, or animal subjects. As a consensus- based project involving expert panelists only, formal ethical approval was not required according to institutional and national regulations. All methods were performed in accordance with the relevant guidelines and regulations, and all participants provided written informed consent before taking part in the Delphi process.

## Results overview

A total of 57 statements were initially developed based on a literature review and structured expert discussions. Following the first voting phase, statements that did not reach the predefined acceptance level of cumulative agreement, were revised according to panel feedback and submitted for further evaluation. In addition, some statements were merged to improve clarity and reduce redundancy. Following the second voting phase and the subsequent discussion, three additional statements related to surgical management were developed and submitted for voting. The final evaluation included 49 unique statements spanning several thematic areas.

All statements and their respective level of agreement are presented in Tables 1, 2, 3, 4 and 5.

**Table 1:** Statements voted on topics: selection of patients to be included in the diagnostic and monitoring process; NF1-associated PNs diagnosis

*Selection of patients to be included in the diagnostic and monitoring process*	*Agreement %*	*References*
A PN, although apparently isolated, requires further diagnostic investigations for NF1.	100	[6]
All individuals with NF1 should be screened (clinically and/or radiologically) for the presence of PNs.	82	[2, 3, 6, 7, 16]
*NF1-associated PNs diagnosis*	*Agreement %*	*References*
A careful physical examination should be performed at every clinical visit by clinicians with NF1 expertise.	94	[7, 11, 16]
A careful clinical evaluation of PNs should be performed at the time of NF1 diagnosis or when NF1 is clinically suspected.	94	[7, 11, 16]
In case of clinical suspicion, regional MRI should be performed.	82	[7, 11, 16]
In absence of clinical suspicion, a screening MRI study of the entire body (first choice by WB-MRI, if unavailable by segmental MRI) should be considered at least at transition to adulthood.	100	[16, 24, 25]
Clinical appraisal of a PN may be carried out through observation, palpation, and neurological examination; photography or video can be useful adjuncts to these assessments.	94	[16]
In the clinical assessment every possible sign or symptom due to PN should be searched and documented.	100	[6, 7, 22]
In case of symptoms, signs of doubt, at any age, imaging should be performed to document a PN.	82	[7, 16]
Ultrasound (US) can serve as a simple, rapid, and practical method for diagnosing and monitoring superficial small lesions, offering several advantages over MRI, such as lower cost, greater patient compliance (especially in early childhood), and the lack of need for sedation.	76	[27, 29, 31]
Biopsy should be reserved for cases with clinical or radiological suspicion of malignant behavior or transformation. To minimize diagnostic errors, especially in extensive and heterogeneous lesions, multiple biopsy samples should be obtained from distinct areas with different radiological features.	88	[28]

**Table 2:** Statements voted on topics: MRI-based techniques for the assessment and measurment of PNs; MRI-based classification of PNs

*MRI-based techniques for the assessment and measurements of PNs*	*Agreement %*	*References*
Mandatory sequences are: T1-weighted (pre-contrast): Provides detailed anatomical information and delineates fat planes.	88	[6, 7, 30–32]
Mandatory sequences are T2-weighted with fat suppression or STIR (pre-contrast): Enhances lesion conspicuity by highlighting high water content; useful for identifying extent and internal characteristics of PNs.	87	[6, 7, 30–32]
Optional/Advanced Sequences are Post-contrast T1-weighted with fat suppression (Gadolinium-enhanced): Evaluates lesion vascularity and helps detect features suggestive of malignant transformation.	81	[6, 7, 30–32]
Optional/advanced Sequences are Diffusion-weighted imaging (DWI): may help in differentiating benign from malignant lesions (e.g., malignant peripheral nerve sheath tumor, MPNST) by identifying restricted diffusion.	81	[6, 7, 30–32]
One-dimensional (RECIST criteria) or two-dimensional (WHO criteria) measurements on regional MRI are commonly used in clinical practice to evaluate lesion size, growth rate, and treatment response; however, they may not always provide sufficient information, particularly in complex or heterogeneous lesions.	81	[32]
According to the Tumor Measurement Working Group of the REiNS (Response Evaluation in Neurofibromatosis and Schwannomatosis) Committee, regional MRI with volumetric analysis is recommended for the sensitive and reproducible evaluation of tumor size changes in the clinical trial setting.	81	[7, 32]
*MRI-based classification of PNs*	*Agreement %*	*References*
The radiological report should include a description of the PN’s morphology and internal architecture, categorizing it as fascicular multinodular (composed of tubular, spherical, or combined elements), diffuse (lacking a defined geometric pattern), or mixed (a combination of both).	81	[7]
The depth of PNs should be reported in the radiological assessment as deep (located beneath the muscle fascia), superficial (involving subcutaneous or cutaneous tissues), or mixed (a combination of both).	87	[7]
The radiological report should describe the relationship between PNs and adjacent tissues as interdigitated or intricately intertwined, well-demarcated and circumscribed, or mixed (exhibiting features of both).	87	[7]

**Table 3:** Statements voted on topics: MRI characterization of DNLs; Natural History of PNs

*MRI Characterization of DNLs *	*Agreement %*	*References*
Radiologists should be aware of accurately reporting DNLs which to date are underreported.	88	[13, 48]
DNLs may exhibit a growth rate that is independent of age and may have a higher potential for transformation into MPNSTs compared to PNs. As a result, DNLs require a multidisciplinary evaluation to prompt tailored follow up.	100	[7–9, 13]
*Natural history of PNs*	*Agreement %*	*References*
Regardless of the patient age, the growth rate of a PNs should be established after diagnosis through longitudinal follow-up assessments.	100	[6, 7, 33–35]
Growth monitoring should be conducted at shorter intervals in pediatric patients than in adults.	77	[7, 33–35]
Puberty and pregnancy do not typically warrant modifications to the follow-up of a PN.	75	[13, 33, 35–37]

**Table 4:** Statements voted on topics: Morbidity and mortality; Time of surveillance of PNs

*Morbidity and mortality of PNs*	*Agreement %*	*References*
Patients with PN should be aware that even if symptoms are absent at diagnosis, there is a risk they will appear later.	100	[7, 16]
PN-related morbidities should be evaluated with specific exams and validated functional measures where available.	94	[7, 16, 39]
Pain due to PN should be evaluated in terms of intensity and interference with validated scales.	100	[7, 15, 42]
Pain due to the PN is a frequent reason to treat.	88	[10–12, 40, 41]
QoL should routinely be assessed in patients with PN using validated questionnaires.	88	[10, 38, 39, 43, 44]
Psychological evaluation in children and adults with a PN, but also of their parents and caregivers, is recommended to assess its impact on QoL, self-esteem etc., and psychological support should be offered to patients and caregivers.	88	[10, 38, 39, 43, 44]
PNs causing compression, particularly of those vital ones, or spine and/or dislocation of internal organs should be considered likely symptomatic, even in the apparent absence of immediate consequences, and should be evaluated for being treated.	82	[7, 39, 49]
While PN size can be estimated radiologically, its clinical relevance should be interpreted by experienced clinicians who take into account the lesion’s topography, as some anatomical regions are more tolerant than others to larger volumes.	82	[39]
Patients with PN should be made aware of the risk of progression to atypical and malignant lesions, underscoring the need for a self-care education program.	94	[6, 7, 15]
Radiologists should report any concerning sign suggestive of potential progression: sudden increase in size, loss of target sign, infiltrative margins, necrosis or cystic degeneration, restricted diffusion (on DWI), peripheral enhancement with central necrosis.	94	[6, 7, 16]
The onset or worsening of pain, as well as the rapid growth of PNs, should always prompt further diagnostic investigations, including MRI, FDG-PET, biopsy, and/or surgery. The indication, selection of these investigations, and their results should be discussed within a multidisciplinary expert team (MDT).	94	[6, 7, 16, 45]
*Time of PNs surveillance*	*Agreement %*	*References*
The time interval of follow up should be determined on the basis of different aspects including symptoms, clinical examination, patient age, location and characteristics of PNs.	82	[7, 32]
A 12-month follow-up could be proposed for asymptomatic, known, stable lesions, especially in adolescent and adult age.	77	[7, 13, 33, 34]
A 3–6-month follow-up is recommended for growing or newly symptomatic plexiform neurofibromas. However, lesions showing > 20% growth or clinical features suggestive of malignant transformation, require immediate evaluation, as they may need a different diagnostic workup.	82	[7, 13, 33, 35, 48]
An immediate revaluation should be performed for those lesions becoming symptomatic and/or rapidly growing.	94	[7, 16, 35, 48]
Timely suspicion and diagnosis of malignant transformation are critical for improving patient outcomes.	94	[6–9, 13, 48]

**Table 5:** Statements voted on topic: Surgical assessment of PNs

*Surgical assessment of PNs*	*Agreement %*	*References*
In PNs management the term” inoperable” refers to a PN that cannot be completely removed without causing significant harm or complications because of proximity to vital structures, invasiveness, or high vascularity.	79	[49]
The operability of a PN and the indication for surgery should be established by a MDT, including surgeons with both expertise in nerve sheath tumors and familiarity with the anatomical location of the tumor.	94	[7, 16, 23, 49]
Surgery is the preferred treatment in case of symptomatic and safely resectable PNs.	94	[49, 51, 53]
In cases of DNLs with imaging characteristics suspicious for malignancy (e.g., growth rate, MRI, PET features), multidisciplinary discussion is required, and lesion biopsy and/or resection should be considered whenever possible.	94	[7, 33]
Clinical appraisal of some PNs may be carried out through observation, palpation, and neurological examination; photography or video can be useful adjuncts to these assessments.	82	[7, 16]
Surgical debulking may be a reasonable option for selected patients in the integrated treatment of PNs.	82	[49]
The tumor’s location, size, and proximity and relationship to surrounding anatomical structures all impact the extent of surgical radicality.	93	[50–52]
During surgical evaluation, it should be taken into account that residual PN in pediatric patients grow faster than in adults.	93	[13, 26, 33–35]

The approved statements are summarizing below according to thematic domain and are followed by a related discussion.

## Results and discussion

***Statements 1 and 2: ****Selection of patients to be included in the diagnostic and monitoring processes* (Table 1).

In the general population, neurofibromas and schwannomas are the most common benign peripheral nerve sheath tumors. They typically arise in adulthood and usually present as solitary, sporadic lesions. Approximately 95% of isolated neurofibromas and schwannomas occur sporadically [6]. In contrast, PNs are strongly associated with NF1 and are considered a hallmark feature of the disorder, as they rarely occur outside the context of NF1 [6, 7].

Consequently, in cases of an isolated PN, the I-NF1-EP recommended a systematic evaluation for additional diagnostic criteria of NF1, including NF1 genetic testing (germline analysis and, if negative, tumor DNA analysis), particularly in children (100% agreement rate).

While the presence of a PN should always prompt a diagnostic work-up for NF1, all individuals with NF1 should also undergo clinical and/or radiological screening for PNs. However, full consensus was not reached for statement 2 (82% agreement rate), reflecting persisting uncertainty regarding the optimal timing and modalities of screening.

***Statements 3–11: ****NF1-PNs diagnosis* (Table 1).

The I-NF1-EP strongly agreed (94%) that every effort should be made to ensure the early diagnosis of PNs.

Early diagnosis of PNs, achieved through the integration of clinical and radiological assessments, is crucial even in asymptomatic individuals. It allows for the assessment of the risk of future symptom development, supports longitudinal monitoring of tumor growth, and enables the early detection of clinical or imaging features suggestive of malignant transformation.

Evidence indicates that PNs can be clinically identified in approximately 30% of patients with NF1 [21]: they often cause body asymmetry and deformities and are frequently covered by thick, hyperpigmented skin, sometimes associated with hypertrichosis [22, 23].

However, whole-body MRI studies (WB-MRI), enabling the detection of deep-seated PNs, suggest that their true prevalence may reach up to 50% [24–26].

Consequently, the I-NF1-EP strongly agreed (94%) that in cases of confirmed or suspected NF1, particular attention should be paid to clinical history and physical examination, which must be performed by clinicians experienced in NF1 and PNs.

If clinical suspicion of a PN is present, the NF1 specialist proceeds, regardless of patient age, with a request for radiological assessment using segmental MRI (82% agreement rate).

In the absence of clinical suspicion of PNs, screening strategies should be considered, including WB-MRI (or at least brain and spine MRI) at the time of transition to adulthood (100% agreement).

The I-NF1-EP strongly agreed with the European Reference Network (ERN) for Genetic Tumor Risk Syndromes (GENTURIS) tumor surveillance guidelines for individuals with NF1, which were developed based on expert consensus [16]. These guidelines recommend clinical assessment for PN at every clinical visit, starting from birth or diagnosis in all individuals with NF1. Although there is no consistent evidence from the literature, WB-MRI is recommended during the transition from childhood to adulthood (i.e., between 16 and 18 years) to assess the internal tumor burden and develop a surveillance plan for MPNST risk.

Although MRI remains the gold standard for the diagnosis of PNs, the authors acknowledged that ultrasound (US) should be considered an adjunct imaging modality for the assessment of superficial lesions [27]. However, the consensus on its use was relatively low (76% agreement rate), underscoring the need to better define its utility and limitations. As a relatively low-cost modality, US may support the initial exclusion of potential mimicking pathologies and, when clinically indicated, provides guidance for percutaneous biopsy [28]. Importantly, US should be performed by operators with specific expertise in NF1 and PNs, as its reliability is highly operator-dependent [6, 29].

Suspected malignant transformation should be evaluated by a multidisciplinary team (MDT) including sarcoma specialists. When biopsy is indicated, multiple samples are recommended to capture tumor heterogeneity and avoid missing malignant regions (88% agreement rate).

***Statements 12–17: ****MRI-based techniques for the assessment and measurement of PNs* (Table 2).

The multidisciplinary expert panel recognized the need to standardize MRI protocols for PN diagnosis and characterization, although consensus was not unanimous, as some clinicians felt insufficiently confident to comment on the radiological protocol.

The gold-standard imaging modality for PNs is regional or whole-body MRI, including T1-weighted (88% agreement rate), T2-weighted with fat suppression, and short tau inversion recovery (STIR) sequences (87% agreement rate). These can be performed without intravenous contrast and provide optimal visualization of the extent and characteristics of PNs by highlighting their high-water content.

Post-contrast T1-weighted images with fat suppression and diffusion-weighted sequences are considered advanced techniques useful for assessing PN vascularity and detecting features suggestive of malignant transformation (81% agreement rate). Therefore, contrast administration should be reserved for cases with suspected malignant transformation.

PNs are typically isointense to muscle on T1-weighted images and show heterogeneous high-signal intensity on T2-weighted sequences. The target sign, defined as central hypointensity and peripheral hyperintensity on T2, diffusion, and post-contrast T1-weighted MRI sequences, is suggestive of a benign lesion [30]. Enhancement is usually moderate and variable after contrast agent administration [31].

One-dimensional (RECIST criteria) and two-dimensional (WHO criteria) measurements on regional or whole-body MRI may be sufficient to assess lesion size, growth rate, and treatment response in clinical practice; however, the panel agreed that these methods may be inadequate for complex or heterogeneous lesions (81% agreement rate). According to the Tumor Measurements Working Group of the Response Evaluation in Neurofibromatosis and Schwannomatosis (REiNS) Committee, MRI with volumetric analysis is the most sensitive and reproducible method for evaluating tumor size and is recommended in clinical trials (81% agreement rate) [32]. However, broader adoption of three-dimensional (3D) imaging in NF1 is hindered by high cost, limited availability of specialized expertise, and software constraints that can compromise the accuracy of volumetric measurements.

***Statements 18–20*: ***MRI-based classification of PNs* (Table 2).

The I-NF1-EP opted to adopt the radiological classification of PNs recently proposed by a multidisciplinary panel of American experts (81–87% agreement rate) [7]. Although this classification does not include all types neurofibromas, such as paraspinal lesions or those with a streaky appearance, it is considered useful in clinical practice to standardize PNs description.

On MRI, based on morphology, a PN can present as a diffuse type with a homogeneous internal structure, a complex multinodular mass composed of irregular/serpentine structures (“bag of worms” appearance), or a mixed mass. Based on depth, a PN can be considered deep when located beneath the muscle fascia, superficial, or mixed. With respect to adjacent structures, PN margins may be well-defined, poorly defined or mixed [7].

***Statements 21 and 22***: *MRI Characterization of Distinct Nodular Lesions* (Table 3).

The I-NF1-EP emphasized the need for radiologists to accurately report the presence of DNLs, which are not always adequately documented in radiology reports in clinical practice (88% agreement rate).

Recent literature has introduced this terminology to describe specific peripheral nervous system tumors that, in patients with NF1, develop within or outside a PN. DNLs are well-demarcated lesions with a diameter of at least 3 cm and lack the characteristic target sign observed in classic nodular PNs. Compared to classic PNs, DNLs in young patients exhibit a faster growth pattern, with a median growth rate of approximately 28% per year [13]. However, unexpectedly, a recent long-term follow-up study evaluating the growth of 324 internal neurofibromas in 47 adults found that, of the 29 DNLs identified, only 13.8% showed growth over time, whereas 62.1% shrank spontaneously by at least 20% over a decade [33]. Overall, the current findings indicate that the radiologic detection of a DNL may serve as a predictor of growth in children, whereas this association does not appear to exist in adults [33].

DNLs have been proposed to represent PNs with more aggressive behavior because of their rapid growth rate in children, association with elevated SUVmax, and distinctive histologic features [9, 13]. DNLs may represent the imaging correlate of atypical neurofibromatous neoplasms of uncertain biological potential (ANNUBP). This recently described entity is characterized by at least two of the following features: cytological atypia, hypercellularity, loss of neurofibroma architecture, and an increased mitotic index. Along with biallelic NF1 inactivation, a recurrent loss of the CDKN2A/B locus at 9p21.3 has been identified.

ANNUBPs are now considered premalignant lesions, as they represent an intermediate stage between PN and MPNST [14]. Although further studies are needed to better define the biological significance and natural history of DNLs, radiologists should accurately identify and properly report them in radiology reports. Clinicians must be aware that DNLs require multidisciplinary evaluation to ensure personalized follow-up and timely surgical resection (100% agreement rate).

***Statements 23–25: ****Natural history of PNs* (Table 3).

The observation that many PNs are diagnosed in early childhood suggests that they are either present at birth or arise during the first years of life [7, 22].

The PN growth rate varies not only among individuals but also between lesions within the same patient; however, the growth rate of an individual PN appears to remain relatively constant over time [7]. In general, the median growth rate of PNs in children and young adults ranges from 3.7% to 12.4%−14.3% per year [13, 34, 35]. In a natural history study involving 92 pediatric NF1 patients, 76% exhibited a volume increase of 20% or more over three years, with 41 patients showing a median annual volume growth rate exceeding 15% [10]. With regard to the proportion of PNs exhibiting significant growth (≥ 20% per year), Nguyen et al. reported that 13.5% of 71 PNs showed significant growth, with 70% of the growing tumors occurring in patients younger than 18 years [35]. In contrast, PN typically grows more slowly in adolescents and adults, with fewer than 5% of patients experiencing an annual growth rate of ≥ 20% [7]. Spontaneous PN shrinkage has rarely been observed in children [10], whereas it appears to occur more frequently in adults [33].

Indeed, a recent long-term WB-MRI follow-up study of 47 adults with NF1 (median age at baseline WB-MRI: 42 years) evaluated 324 internal neurofibromas over a median follow-up period of 10.4 years. Overall, 62.8% of lesions decreased spontaneously in volume without treatment, whereas 17.1% showed growth over time. When analyzed according to lesion subtype, spontaneous shrinkage was observed in 56% of PNs and 62.1% of DNLs, while growth occurred in 17.9% of PNs and 13.8% of DNLs [33]. Taken together, these data emphasize the need to assess PN growth trajectories for clinical management and clinical decision-making (100% agreement rate). Any PN showing accelerated growth relative to its previous trajectory or an increase of more than 20% within one year, regardless of patient age, should raise suspicion of possible malignant transformation. Natural history findings justify stricter surveillance in children and adolescents than in adults (77% agreement rate), regardless of hormonal changes such as puberty, pregnancy, or contraceptive use (75% agreement rate) as risk factors for PN growth have not been identified [33, 36, 37]. Consensus on these two final statements was borderline: the first was considered appropriate but too generic, whereas the second was deemed insufficiently supported by the literature.

***Statements 26–36*: ***Morbidity and mortality of PNs* (Table 4).

The statements in this domain first addressed the clinical effects of PNs.

At the time of diagnosis, approximately one-third of PNs are already symptomatic, whereas initially asymptomatic tumors may become symptomatic over time [7, 38]. The panel unanimously agreed that patients should be made aware of this possibility (100% agreement rate), as this information may help them recognize relevant symptoms and seek timely medical evaluation. Clinical manifestations are highly variable and depend on patient age, tumor size, location, nerve involvement, and the effect of the tumor on adjacent vital structures.

In children, over 60% of symptomatic PNs are located in the head and neck, whereas tumors in the thorax and abdomen frequently remain silent. Conversely, adults with PNs involving the brachial or lumbosacral plexus, or the abdominopelvic region are at particularly high risk of developing significant morbidity [7, 39]. In addition to size, topography appears to be important, as some anatomical regions are more tolerant than others of large tumor volumes (i.e. the thorax versus the eyelid) (82% agreement rate).

Pain is the most common symptom associated with PNs in both children and adults and represents one of the main reasons for initiating treatment (88% agreement rate), given its detrimental effect on daily functioning and QoL. Its clinical relevance has been documented in natural history studies and further underscored by reports of pain improvement following treatment with MEK inhibitors [10–12, 40, 41]. Moreover, pain may worsen over time in patients with progressive tumor growth.

Consequently, the panel unanimously agreed that PN-related pain should be routinely assessed at diagnosis and longitudinally thereafter, taking into account its intensity, frequency, and interference with daily activities. The selection of assessment tools should be guided by patient age and clinician expertise, as no universally validated instrument specifically designed for PN-related pain currently exists [42].

Finally, given that worsening of pain or pain refractory to treatment may indicate malignant transformation, the panel strongly agreed (94% agreement rate) that patients with PNs should be informed about this risk and educated about the warning signs of disease progression.

While pain and disfigurement are the most frequently reported symptoms across all age groups, patients may also suffer from motor dysfunction, sensory impairments such as vision or hearing loss, airway obstruction, and difficulties with speech, swallowing, breathing, bowel, and bladder function. Local skin changes in some PNs, such as thickening, hyperpigmentation, or hypertrichosis, further emphasize the visibility of the tumor.

Ultimately, all symptoms have a profound negative impact on QoL by restricting daily activities, lowering self-esteem, and causing emotional disturbances that significantly impair social relationships [43, 44].

Consequently, children, as well as their parents and caregivers, and adults with PNs should undergo routine assessments of QoL, self-esteem, and related aspects, using validated questionnaires (88% agreement rate). While there is general agreement that psychological support should be offered to both patients and caregivers, in Italy this is not always feasible within the resources of the national healthcare system.

Ideally, PN-related symptoms should be monitored longitudinally using standardized and reproducible measures (94% agreement rate); however, their routine use in clinical practice may be challenging because of the time required and the considerable heterogeneity of symptom presentation.

Nevertheless, regular monitoring of symptoms and their response to treatment remains crucial, as symptom improvement is the primary goal of PN management. Moreover, symptom improvement may occur even in the absence of a significant volumetric response.

The panel agreed that PNs causing compression, encasement, or displacement of critical structures should be carefully evaluated for treatment, even when asymptomatic, as these lesions have the potential to cause significant symptoms as they grow (82% agreement rate).

Mortality in the NF1 population is primarily driven by malignant transformation of PNs and by life-threatening compressive complications including airway obstruction and spinal cord compression.

Patients with NF1 develop MPNSTs at a significantly younger age than those without NF1 (mean age 28 vs. 41 years) and experience substantially poorer survival, with a 5-year survival rate of 16–32% compared with 33–51% in the general MPNST population. The risk of malignancy is increased in carriers of large deletions encompassing the *NF1* gene [45] and missense variants affecting codons 844–848 [46]. Zhu et al. recently identified further genotype–phenotype associations in NF1, reporting a higher prevalence of spinal PNs among individuals with splicing variants and an increased malignancy risk associated with the recurrent p.Arg1748 variant [47]. Although these findings require confirmation in larger patient cohorts, they further highlight the unmet need to stratify the risk of PN and MPNST in NF1 patients based on genotype, that is currently possible in only approximately 10% of patients.

ANNUBPs are at higher risk for the development of MPNST, with a 33% incidence in one study versus the cumulative MPNST risk of 15.8% in the overall NF1 population [9, 48].

Malignant progression is usually associated with a more rapid growth rate, changes in MRI characteristics, worsening or appearance of spontaneous pain, and eventual abnormalities of the covering skin (e.g. ulceration, redness, etc.). The panel emphasized the importance of early recognition of these transformation indicators (94% agreement rate). The techniques potentially useful for evaluating malignancy were identified as MRI, FDG-PET, biopsy, and/or surgery. The selection of the most appropriate diagnostic approach should be determined by a MDT that includes a clinician and a surgeon with expertise in sarcomas and is responsible for reviewing and discussing the results (94% agreement rate).

It is reasonable to hypothesize that combining early MRI detection of PNs and/or DNLs with close MRI follow-up could enable timely identification of malignant transformation and ultimately reduce mortality rates [48].

***Statements 37–41*: ***Timing of PN surveillance* (Table 4).

Follow-up timing should be individualized according to multiple parameters including symptomatology, clinical examination findings, patient age, anatomical location, and imaging characteristics of the PN.

The experience of both the disease manager and the radiologist is pivotal in determining appropriate surveillance intervals.

Indeed, despite growing evidence that close monitoring and management of PNs and DNLs have significant implications for patient outcomes, standardized surveillance protocols are currently lacking [48].

To facilitate implementation in clinical practice, the panel developed a pragmatic framework summarizing the suggested MRI follow-up intervals across the most common clinical scenarios. Lesions associated with pain, new symptoms, or unexpected growth should undergo prompt clinical and radiological assessment (94% agreement rate). MRI surveillance every 3–6 months is recommended for newly diagnosed, symptomatic or growing PNs (82% agreement rate), whereas an annual MRI evaluation may be appropriate for known, asymptomatic, and radiologically stable PNs (Table A). However, consensus regarding annual surveillance was less robust (76% agreement rate), reflecting the need for individualized follow-up based on lesion characteristics and clinical context. DNLs should be managed through individualized and multidisciplinary assessment.

**Table:** Suggested timing for surveillance of PNs and DNLs independently by the patient’s age

Clinical scenario	Suggested MRI interval
Newly diagnosed PN	3–6 months
Symptomatic PN	3–6 months
Growing PN	3–6 months
Stable asymptomatic PN	12 months
DNL	Individualized MDT follow-up
Suspected malignant transformation	Immediate work-up

***Statements 42–49*: ***Surgical assessment of PNs* (Table 5).

Until recently, surgery, together with symptomatic treatment, was the only available therapeutic option for symptomatic PNs.

Nevertheless, evidence supporting surgical management remains limited, with few systematic data on indications, clinical outcomes, treatment-related morbidity, and recurrence rates.

Current evidence supports surgery as the treatment of choice for symptomatic PNs whenever complete resection can be achieved without significant functional consequences (94% agreement rate) [7, 49].

In practice, however, complete resection is rarely possible because of the infiltrative nature of these tumors, their hypervascularity, poorly defined margins, and proximity to vital structures. Indeed, complete resection is achieved in only about 10% of elective pediatric procedures, while residual disease, persistent symptoms, and procedure-related complications remain frequent, particularly in patients with head and neck involvement [50]. In addition, less extensive resections are associated with shorter intervals between procedures, particularly for tumors of the head, neck, and thorax which show the highest recurrence rates [51, 52]. Consistent with the natural history of PNs, tumor regrowth occurs in 20%−68% of cases, with younger age (≤ 21 years), tumor location, and tumor depth representing the main predictors of progression [26].

Consequently, surgical decision-making is complex and highly individualized. The panel agreed that patients with PNs should be evaluated by a MDT including clinicians, oncologists, and surgeons experienced in nerve sheath tumors and in the involved anatomical region (94% agreement rate). Depending on tumor location, the contribution of multiple surgical specialists may also be required.

Current indications for surgery include DNLs and lesions suspicious for malignant transformation, progressive tumor enlargement, significant functional impairment, and intolerable sensory symptoms [7, 49].

The indication for surgery in asymptomatic, small, and superficial PNs that are amenable to complete resection remains controversial. Although asymptomatic, their surgical removal may help prevent further progression affecting appearance and functionality [53].

A key point of the discussion concerned the concept of PN inoperability, as treatment with MEK inhibitors is currently authorized only for patients with inoperable PNs.

Given that most PNs are technically amenable to surgery, the panel agreed that, in this context, the term ‘inoperable’ refers to a lesion that cannot be completely resected without causing substantial morbidity or complications, because of its proximity to vital structures, infiltrative growth pattern, or high vascularity (78.5% agreement rate). However, it was difficult to identify a universally accepted definition.

The panel also acknowledged that partial resection of a PN is associated with a risk of regrowth, especially in younger patients [26]. This risk has important implications for surgical outcomes and should be carefully considered during the decision-making process (93% agreement rate). The statements also supported standardized preoperative assessments, including detailed MRI, functional studies, photography or video, and evaluation of patient-specific goals (e.g., pain control, disfigurement reduction, cosmetic improvement, motor recovery) (82% agreement rate). Postoperative follow-up is essential for detecting early signs of recurrence or complications.

Moreover, with the advent of new pharmacological therapies, the role of surgery in PN management has broadened and should be considered within a multidisciplinary treatment strategy. In selected cases, surgery may be used in combination with systemic therapy for either neoadjuvant or adjuvant purposes, especially for large lesions or PNs involving critical anatomical sites (82% agreement rate).

### Role of MEK inhibitors in the current management of PNs

The introduction of MEK inhibitors has profoundly changed the management of NF1-associated PNs providing an effective therapeutic option for patients with symptomatic and inoperable PNs, and demonstrating clinically meaningful improvements in tumor volume, pain, function, and QoL. The concept of inoperability remains challenging, as also highlighted by our results, and should be assessed within a multidisciplinary setting. In many cases, lesions are technically resectable, but complete surgery would be associated with unacceptable morbidity or major functional impairment. In such situations, treatment with MEK inhibitors may represent the preferred initial therapeutic strategy. The availability of targeted therapies also supports a more integrated medical-surgical approach, in which surgery and systemic treatment should not be considered mutually exclusive but rather complementary options tailored to the individual patient and tumor characteristics. Future consensus initiatives specifically focused on medical therapies will be essential to better define the optimal integration of these agents into the multidisciplinary management of NF1-associated PNs.

Nevertheless, the expert panel deliberately chose not to address pharmacological treatments in detail within the scope of the present consensus. The rapidly evolving therapeutic landscapes, including issues related to treatment indications, duration, toxicity management, sequencing with surgery, and long-term outcomes, warrants a dedicated discussion beyond the objectives of this work.

### Importance and clinical implications of the study

This study presents the results of a national multidisciplinary consensus specifically dedicated to the lifelong management of PNs in patients with NF1. In a clinical landscape profoundly reshaped by the introduction of MEK inhibitors, this work addresses several areas of uncertainty commonly encountered in routine clinical practice. Using a RTD methodology, NF1 specialists translated the available evidence and their collective clinical experience into practical recommendations addressing PN diagnosis, surveillance, surgical assessment, and the integration of medical and surgical treatment within a multidisciplinary framework.

The present consensus should be regarded as complementary to the ERN GENTURIS surveillance guidelines. While ERN GENTURIS provides overarching recommendations for surveillance in NF1, our work focuses specifically on PN management and offers more detailed guidance on several issues that remain incompletely defined in current recommendations, including MRI acquisition and reporting, characterization of DNLs, surveillance intervals, criteria for surgical referral, and the integration of surgery and targeted therapies.

Overall, the panel’s recommendations are consistent with the general principles outlined by Fisher et al., and no major areas of disagreement were identified. Rather than diverging from existing recommendations, this consensus refines and operationalizes them by providing practical guidance in areas where evidence remains limited and clinical practice remains heterogeneous.

The multidisciplinary composition of the panel, the use of a RTD methodology, and the integration of current evidence with extensive clinical expertise represent important strengths of this initiative. The resulting framework may support greater standardization of PN management across Italy while also serving as a model for future consensus-building efforts in other healthcare systems.

### Limitations of the study

Some limitations should be acknowledged. First, although consistent with RTD methodology and the highly specialized nature of the topic, the expert panel was relatively small. While this facilitated in-depth discussion among experts, it may have limited the diversity of perspectives included in the consensus process.

Second, all participating experts were based in Italy. Consequently, the resulting recommendations may reflect country-specific clinical practices, healthcare organization, and referral pathways, potentially limiting the generalizability of the findings to other healthcare systems and international settings.

In addition, the expert selection process may have introduced selection bias. Although panelists were recruited from high-volume referral centers with extensive experience in the management of both pediatric and adult patients with NF1-associated PNs, and efforts were made to ensure broad geographical and multidisciplinary representation, the possibility of selection bias cannot be completely excluded. The inclusion of surgeons and radiologists identified through established multidisciplinary collaborations may have further contributed to this limitation.

Furthermore, although PAGs were consulted during the development and discussion of the consensus statements and provided valuable input from the patient and caregiver perspective, patients did not directly participate in the RTD voting process. As a result, patient-reported priorities and preferences may not have been fully captured in the final recommendations.

Finally, a formal grading system for the quality of evidence and strength of recommendations was not applied. Consequently, some statements were based primarily on expert opinion and collective clinical experience in areas where high-quality evidence remains limited.

## Conclusions

**Fig. 2:**
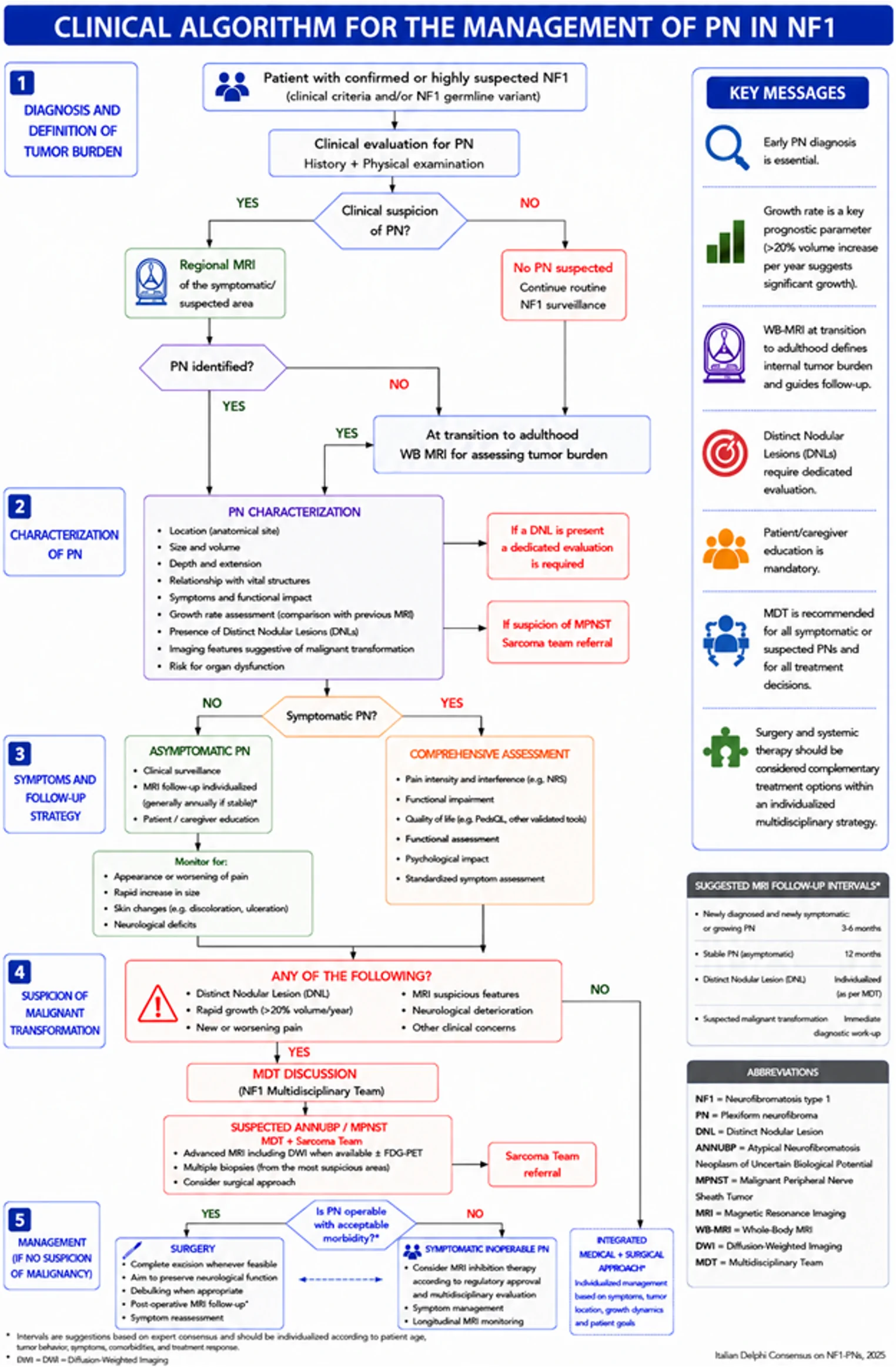
Clinical algorithm for the management of PN in NF1

The development of the first national consensus in Italy on the management of PNs in NF1 represents a major step forward in the care of patients with NF1, which remains fragmented and heterogeneous across regions, hospitals, and medical specialties. This initiative has brought together experts from multiple disciplines, enabling the integration of complementary perspectives and expertise. The resulting consensus provides a practical, evidence-informed framework to support clinicians in the management of NF1-associated PNs. By combining a validated consensus methodology with a digital and anonymous platform, it also represents an efficient and scalable model for generating actionable clinical recommendations that may be applicable beyond the Italian healthcare setting. The high level of agreement achieved across several thematic areas reflects the strength of the available literature and accumulated real-world clinical experience. Nevertheless, important unmet needs remain, particularly regarding surgical management, follow-up schedules, and screening strategies. These gaps, within a clinical landscape reshaped by the approval of novel targeted therapies, warrant further investigation and should be prioritized in future research efforts. To facilitate the implementation of the consensus recommendations in routine clinical practice, a flow-chart summarizing the diagnostic and therapeutic pathway for NF1-associated PNs has been developed, providing clinicians with a concise and practical decision-support tool (Fig. 2).

Future studies and international consensus initiatives will be important to validate and further refine these recommendations as additional evidence becomes available.
